# The MELFO-Study: Prospective, Randomized, Clinical Trial for the Evaluation of a Stage-adjusted Reduced Follow-up Schedule in Cutaneous Melanoma Patients—Results after 1 Year

**DOI:** 10.1245/s10434-016-5263-7

**Published:** 2016-05-18

**Authors:** Samantha Damude, Josette E. H. M. Hoekstra-Weebers, Anne Brecht Francken, Sylvia ter Meulen, Esther Bastiaannet, Harald J. Hoekstra

**Affiliations:** 1 Department of Surgical Oncology, University of Groningen, University Medical Center Groningen, Groningen, The Netherlands; 2 Wenckebach Institute, University of Groningen, University Medical Center Groningen, Groningen, The Netherlands; 3 Netherlands Comprehensive Cancer Organisation, Groningen, The Netherlands; 4 Department of Surgical Oncology, Isala, Zwolle, The Netherlands; 5 Department of Dermatology, Netherlands Cancer Institute/Antoni van Leeuwenhoek, Amsterdam, The Netherlands; 6 Department of Surgical Oncology, University of Leiden, University Medical Center Leiden, Leiden, The Netherlands

## Abstract

**Background:**

Guidelines for evidence-based follow-up in melanoma patients are not available. This study examined whether a reduced follow-up schedule affects: patient-reported outcome measures, detection of recurrences, and follow-up costs.

**Methods:**

This multicenter trial included 180 patients treated for AJCC stage IB-II cutaneous melanoma, who were randomized in a conventional follow-up schedule group (CSG, 4 visits first year, *n* = 93) or experimental follow-up schedule group (ESG, 1–3 visits first year, *n* = 87). Patients completed the State-Trait Anxiety Inventory, cancer worry scale, impact of events scale, and a health-related quality of life questionnaire (HRQoL, RAND-36). Physicians registered clinicopathologic features and the number of outpatient clinic visits.

**Results:**

Sociodemographic and illness-related characteristics were equal in both groups. After 1-year follow-up, the ESG reported significantly less cancer-related stress response symptoms than the CSG (*p* = 0.01), and comparable anxiety, mental HRQoL, and cancer-related worry. Mean cancer-related worry and stress response symptoms decreased over time (*p* < 0.001), whereas mental HRQoL increased over time (*p* < 0.001) in all melanoma patients. Recurrence rate was 9 % in both groups, mostly patient-detected and not physician-detected (CSG 63 %, ESG 43 %, *p* = 0.45). Hospital costs of 1-year follow-up were reduced by 45 % in the ESG compared to the CSG.

**Conclusions:**

This study shows that the stage-adjusted, reduced follow-up schedule did not negatively affect melanoma patients’ mental well-being and the detection of recurrences compared with conventional follow-up as dictated by the Dutch guideline, at 1 year after diagnosis. Additionally, reduced follow-up was associated with significant hospital cost reduction.

**Electronic supplementary material:**

The online version of this article (doi:10.1245/s10434-016-5263-7) contains supplementary material, which is available to authorized users.

The incidence of cutaneous melanoma is rising in most European countries, probably as a result of increased public awareness, resulting in an increase in thinner melanomas at time of diagnosis since the last two decades.^1^,^2^ Recently, a stabilization in incidence has been reported in Australia and North America.^3^ Due to early detection and improved staging with sentinel lymph node biopsy, the 5-year survival rates reported are 92 % for American Joint Committee on Cancer (AJCC) stage IB and 53 % for stage IIC melanoma patients.^4^ Increasing incidence and improved prognosis have resulted in an increased prevalence of melanoma. Consequently, there are more melanoma patients in clinical follow-up.^5^,^6^

For melanoma, there is currently no consensus on the adequate frequency of posttreatment follow-up visits, and surveillance intervals vary widely worldwide.^7^–^9^ Most contemporary surveillance guidelines recommend intensive follow-up schedules.^10^–^12^ Important determinants for surveillance frequency are patients’ reassurance and anxiety reduction, early detection of recurrences or second primary melanoma, and evaluation of the quality of surgical treatment.^13^^–^17 Patients’ preferences regarding follow-up frequency are understudied. However, mixed feelings have been reported. It seems important to balance patients’ reassurance without inducing additional anxiety.^18^,^19^

The majority of melanoma recurrences and second primary melanomas occur within 3 years after initial treatment, with an increase in occurrence per AJCC stage.^14^,^20^ Approximately 75 % of the recurrences and almost 50 % of the second primaries are detected by patients themselves or their partners instead of by clinicians.^21^,^22^ Patient education might even enlarge the number of patient-based detections of recurrent disease.^23^ This implies that follow-up visits may currently be scheduled more frequently than necessary, possibly needlessly burdening patients and health care resources.^21^,^22^

There is a need for guidelines with an evidence-based follow-up frequency. The melanoma follow-up (MELFO) study was designed to determine whether a stage-adjusted follow-up schedule adversely affects melanoma patients’ mental well-being and the detection of first recurrences or second primary melanomas and whether it decreases yearly costs per patient.

## Methods

### Study Design

This randomized, controlled, multicenter trial was initiated by the University Medical Center Groningen (UMCG), conducted in six hospitals in the Netherlands in accordance with the Declaration of Helsinki, and approved by the central medical ethics committee (METc2004.127). Given the nature of the study, it was not possible to blind participants or physicians/nurse practitioners for group assignment. The conventional follow-up schedule was according to Dutch Melanoma guideline recommendations.^11^ The experimental schedule was defined with an overall reduction of 27 % of the number of conventional schedule visits during the first 5 years after diagnosis, based on the previously reported annual risk of recurrence development per AJCC stage: IB 18.4 %, IIA 28.9 %, IIB 41.0 %, IIC 45.2 % (Table 1).^21^,^24^

**Table 1:** Frequency of follow-up visits for conventional follow-up schedule, recommended by the Dutch melanoma working party, and reduced experimental follow-up schedule

Conventional follow-up schedule							Experimental follow-up schedule						
Years*	1	2	3	4	5	6–10	Years*	1	2	3	4	5	6–10
*AJCC stage*							*AJCC stage*						
IB	4	3	2	2	2		IB	1	1	1	1	1	1
IIA	4	3	2	2	2	1	IIA	2	2	1	1	1	1
IIB	4	3	2	2	2	1	IIB	3	3	2	1	1	1
IIC	4	3	2	2	2	1	IIC	3	3	2	1	1	1

* Years after surgery for primary melanoma

Primary endpoint was patients’ mental well-being. Secondary endpoints were development of recurrence or second primary melanoma, the person detecting it, and total hospital costs.

### Patients and Procedure

All patients diagnosed with AJCC stage IB-II cutaneous melanoma, treated with curative intent between February 2006 and November 2013, were eligible for the study. Exclusion criteria were age <18 and >85 years, inadequate knowledge of the Dutch language, and a history of previous malignancy. AJCC stage IA patients also were excluded, as the Dutch Melanoma guideline recommends only a single follow-up visit after treatment.^11^ Physicians or nurse practitioners performing follow-up informed eligible patients about the trial immediately after diagnosis and asked them to participate. After informed consent was given, randomization was performed into the conventional (CSG) or experimental (ESG) follow-up schedule group, stratified for AJCC stage, in random permuted blocks of four patients, generated by a validated system (Intrialgrator) with the use of a pseudo–random number generator and a supplied seed number. Randomization and data management were performed by the Netherlands Comprehensive Cancer Organization (IKNL). The first questionnaire (at inclusion; T1) and a prestamped return envelope were then sent to the patient’s home address. All patients received oral and written information on melanoma and instructions on self-inspection of skin and lymph node bearing areas.^25^ After 12 months (time point 2; T2), patients completed questionnaires again, excluding those with recurrent disease.

### Instruments

Patients completed sociodemographic questions, two self-designed questions regarding follow-up schedule satisfaction, one on self-inspection, and one on the number of melanoma related visits to the general practitioner (GP). Also, they filled in the following validated patient reported outcome measures (PROMs): (1) the 20-item State-Trait Anxiety Inventory-state version (STAI-S), measuring the transitory emotional condition of stress or tension perceived by respondents.^26^ Higher scores (range 20–80) indicate greater anxiety; (2) the 3-item cancer worry scale (CWS), assessing concerns about developing cancer (again) and their impact on daily functioning.^27^ Higher scores (range 3–12) indicate more concerns; (3) the 15-item impact of event scale (IES), assessing the extent to which people are bothered by memories of a major life-event in terms of intrusion and avoidance.^28^ Higher scores (range 15–75) indicate the presence of more intrusion/avoidance; (4) the mental component summary (MCS) score of the RAND-36, a health related quality of life (HRQoL) questionnaire. The MCS score was standardized with a mean of 50 and a standard deviation of 10.^29^

Surgical oncologists, dermatologists, or nurse practitioners performing follow-up, registered melanoma-related variables, and the actual frequency of melanoma-related follow-up visits in the hospital. Follow-up consisted of a comprehensive patient history and physical examination. Laboratory testing and diagnostic imaging was only performed in patients suspicious for recurrent disease, as appropriate.

Total follow-up costs of the first year were calculated for all participating UMCG-patients. Data were received from the financial administration of the UMCG.

### Statistical Analysis

Power analysis for a two-sided test was performed on the STAI-state score with a power *β* = 0.80 and *α* = 0.05. The purpose was to falsify the nil-hypothesis: no difference in STAI-state anxiety between patients in the ESG and the CSG. A sample size of 89 patients in each group was required to prove a difference between the groups of a minimum of 4 points (norm 36.5, standard deviation 9.4). The effect size of this outcome is 0.42.

Statistical analyses were performed on the questionnaires and physician/nurse-practitioner reports after 1 year of follow-up, using IBM SPSS statistics version 22 (SPSS Inc, Chicago, IL). Patient characteristics were compared between the groups using *t* tests and Chi square tests as appropriate. Repeated measures ANOVAs were used to examine differences between study groups in PROMs, change over time, and interaction effects. Effect sizes (ES) were calculated to examine if significant differences found were clinically relevant. ES <0.2 were considered negligible, those between 0.2 and 0.49 small, those between 0.50 and 0.79 moderate, and those ≥0.80 large.^30^ Statistical significance was achieved at *p* < 0.05.

## Results

### Patients

**Fig. 1:**
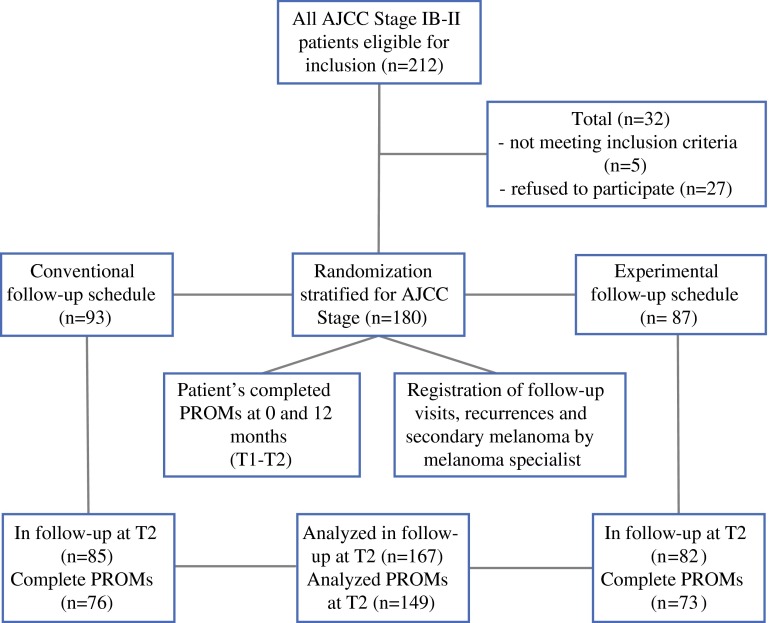
Flow diagram of inclusion and randomization

Of the 212 patients approached, 5 were not eligible and 27 refused participation (response 87 %). A total of 180 patients were randomized, 93 patients were allocated to the CSG, and 87 patients to the ESG (Fig. 1). Sociodemographic and clinicopathologic characteristics were comparable between groups. Median age was 57.4 years, 51.7 % were females, 37.8 % had completed high education (high vocational education or university), 84.4 % had a partner, 47.2 % had paid employment, and 38.9 % reported other co-morbidity. Median Breslow thickness was 1.6 mm. The trunk was more commonly affected in males (54.0 %) and the lower limbs in females (40.9 %, *p* < 0.001). At 1 year after enrollment (T2), 84.5 % of the CSG and 94.2 % of the ESG reported being satisfied with the assigned schedule (*p* = 0.60). Eight CSG patients preferred less frequent follow-up, whereas three CSG and four ESG patients desired more frequent follow-up (*p* = 0.02). Fifteen patients had a recurrence: six before T2 and nine just after T2 questionnaire completion (Table 2).

**Table 2:** Baseline characteristics (CSG: *n* = 93, ESG: *n* = 87) and follow-up related questions; comparison between study groups

Characteristics	Conventional schedule		Experimental schedule		*p* value
	No.	%	No.	%	
Gender					
Female	42	45.2 %	51	58.6 %	0.071*
Male	51	54.8 %	36	41.4 %	
Age (year)					
Median, range	55, 23–81		61, 20–85		0.285^
Level of education^a^					
High	37	39.8 %	31	35.6 %	0.524*
Intermediate	38	40.9 %	33	37.9 %	
Low	18	19.4 %	23	26.4 %	
Relationship status					
With partner	76	81.7 %	76	87.4 %	0.297*
Without partner	17	18.3 %	11	12.6 %	
Daily activities					
Employed for wages	49	52.7 %	36	41.4 %	0.129*
Not employed for wages	44	47.3 %	51	58.6 %	
Presence of co-morbidities					
No	62	66.7 %	48	55.2 %	0.114
Yes	31	33.3 %	39	44.8 %	
Primary melanoma site					
Lower extremity	32	34.4 %	23	26.4 %	0.517*
Upper extremity	17	18.3 %	15	17.2 %	
Trunk	34	36.6 %	41	47.1 %	
Head/neck	10	10.8 %	8	9.2 %	
Breslow thickness (mm)^b^					
Median, range	1.6, 0.3–8.0		1.7, 0.6–7.4		0.733^
<1.00	3	3.2 %	9	10.3 %	0.181*
1.00–2.00	56	60.2 %	42	48.3 %	
2.00–4.00	26	28.0 %	28	32.2 %	
>4.00	8	8.6 %	8	9.2 %	
Ulceration					
No	72	77.4 %	64	73.6 %	0.547*
Yes	21	22.6 %	23	26.4 %	
AJCC stage					
Ib	56	60.2 %	47	54.0 %	0.820*
IIa	19	20.4 %	19	21.8 %	
IIb	12	12.9 %	15	17.2 %	
IIc	6	6.5 %	6	6.9 %	
Schedule satisfaction^c^ *(T2)*					
Yes	60	84.5 %	65	94.2 %	0.064*
No	11	15.5 %	4	5.8 %	
Missing	14		13		
Reason for schedule dissatisfaction^c^					
Wish for less frequent visits	8	72.7 %	0	0.0 %	0.016**
Wish for more frequent visits	3	27.3 %	4	100.0 %	
Frequency of self-inspection^c^ *(T2)*					
At least once a month	58	78.4 %	48	65.7 %	0.232*
Every 3 months	10	13.5 %	16	21.9 %	
Less than every 3 months	6	8.1 %	9	12.3 %	
Missing	11		9		
No. of outpatient clinic visits *(T2)*					
Median, range	4, 2–6		2, 1–4		**0.001**
Less than planned:	10	11.8 %	3	3.7 %	0.051*
−1 visit	8	9.4 %	1	1.2 %	
−2 visits	2	2.4 %	2	2.4 %	
According to assigned schedule	63	74.1 %	60	76.9 %	
More than planned:	12	14.1 %	19	23.2 %	0.133*
+1 extra visit	8	9.4 %	17	21.3 %	
+2 extra visits	4	4.7 %	2	2.5 %	
Reasons extra outpatient clinic visit					
Physical symptoms	9	56.3 %	11	52.4 %	0.956*
Anxiety	6	37.5 %	9	42.9 %	
Other	1	6.2 %	1	4.7 %	
Extra GP consultations^c^ *(T2)*					
No	68	80.0 %	71	86.6 %	0.255*
Yes	17	20.0 %	11	13.4 %	
1 Melanoma-related visit	16	18.8 %	10	12.2 %	0.498*
2 Melanoma-related visits	1	1.2 %	1	1.2 %	
Total extra visits T2 (hospital + GP)					
1 Extra visit	20	23.5 %	19	23.3 %	0.930*
2 Extra visits	5	5.9 %	4	4.9 %	
3 Extra visits	1	1.2 %	2	2.4 %	
Recurrence					
Total	8	8.6 %	7	8.0 %	0.893*
Locoregional	1	12.5 %	0	0.0 %	
In transit	1	12.5 %	1	14.3 %	
Regional lymph nodes	2	25.0 %	2	28.6 %	
Distant	3	37.5 %	1	14.3 %	
Second primary melanoma	1	12.5 %	3	42.9 %	
Detection of recurrence					
Patient	5	62.5 %	3	42.9 %	0.447*
Specialist/NP	3	37.5 %	4	57.1 %	
Cause of death					
Other cause	1	1.1 %	1	1.2 %	0.522**
Melanoma-related^d^	2	2.2 %	1	1.2 %	
Hospital costs (1 year, UMCG)	*n* = 41		*n* = 38		
Total (in Euros), based on:	€ 31,240.67		€ 15,871.11		
Follow-up visits	€ 20,325.88		€ 11,127.17		
By NP	€ 141.20	*n* = 4	€ 176.50	*n* = 5	
By specialist	€ 18,427.21	*n* = 175	€ 8,873.65	*n* = 83	
Telephone consultation	€ 1,757.47	*n* = 22	€ 2,077.02	*n* = 26	
Diagnostics	€ 6,651.91		€ 1,349.67		
Laboratory testing	€ 318.09	*n* = 2	–	–	
Ultrasonography	€ 729.66	*n* = 5	€ 228.40	*n* = 1	
CT-scan	€ 836.89	*n* = 4	–	–	
PET/CT-scan	€ 2,468.83	*n* = 2	–	–	
Bone scan	–	–	€ 344.18	*n* = 1	
Pathology: biopsy/cytology	€ 2,298.44	*n* = 17	€ 777.09	*n* = 7	
Surgery	€ 4,262.88		€ 3,394.27		
Melanoma related	€ 1,424.25	*n* = 4	€ 2,167.44	*n* = 2	
Benign skin lesion	€ 2,838.63	*n* = 5	€ 1,226.83	*n* = 4	
Total per patient, mean ± SD	€761.97	±683.37	€ 417.66	±452.74	**0.010**^

*AJCC* American Joint Committee on Cancer; *GP* general practitioner; *NP* nurse practitioner; *UMCG* University Medical Center Groningen; *T2* after 1-year follow-up

T2: 167 patients included in analyses (CSG: *n* = 85, ESG: *n* = 82)

^a^Highest level of education completed (high: high vocational education, university; intermediate: secondary vocational education, high school; low: elementary school, low vocational education)

^b^Categories based on the publication of Hollestein et al. 31

^c^Self-designed questions

^d^Also included in the number of recurrences

Level of significance *p* < 0.05, printed in bold. * *χ* ^2^ test, ^ Independent student *t* test, ** cell count too low to perform valid *χ* ^2^ test

A total of 19 patients (CSG: 11.8 %, ESG: 9.2 %, *p* = 0.92) were lost to follow-up at T2. Before T2, six patients had recurrent disease (of whom 3 died), and two died of nonmelanoma-related causes. Eleven patients withdrew from the study before T2 because of dissatisfaction with the allocated schedule (CSG: *n* = 5, ESG: *n* = 3) or continuation of follow-up in another clinic (CSG: *n* = 1, ESG: *n* = 2). Excluding these 11 patients plus the 2 deceased of other cause, but including all 15 recurred patients, a total of 44 patients (26.3 %) did not adhere completely to the assigned follow-up schedule. Thirteen patients (7.8 %; CSG: *n* = 10, ESG: *n* = 3) attended less outpatient clinic visits than planned, whereas 31 patients (18.6 %; CSG: *n* = 12, ESG: *n* = 19) paid extra visits, due to melanoma-related anxiety or physical complaints (no significant difference between groups, *p* = 0.068). Besides outpatient clinic visits, some patients also reported melanoma-related visits to the GP. Summarizing outpatient clinic and GP visits, 26 patients (30.6 %) in the CSG and 25 patients (30.5 %) in the ESG paid extra visits during the first year after diagnosis, with a range of one to three extra visits per patient (Table 2). Adherence to schedule was not related to T2 schedule satisfaction. A comparable percentage of satisfied patients (20.5 %, 25/122; CSG: 6 less, 7 extra, ESG: 12 extra) and dissatisfied patients (26.6 %, 4/15; CSG: 1 less, 2 extra, ESG: 1 extra) did not adhere to the schedule as planned.

### Patient Reported Outcome Measures

Of the participants, 83 % completed all questionnaires at T1 and T2 (CSG: *n* = 76, ESG: *n* = 73). PROMs were analyzed for these 149 participants. Repeated measures ANOVAs showed one significant between-group effect: the ESG had significantly lower mean scores on the IES than the CSG (*p* = 0.01). The effect size was small (ES = 0.36). Significant time effects were found on the CWS, IES, and RAND-36 MCS scores (*p* = 0.001). Patients’ CWS and IES mean scores decreased over time, and the RAND-36 MCS score increased over time. Effect sizes were small (CWS and RAND-36: ES = 0.41) and moderate (IES: ES = 0.53). No significant interaction effects were found (Table 3).

**Table 3:** Descriptives of patient-reported outcome measures at baseline (T1) and 1 year (T2), comparison over time and between study groups

Questionnaire	Study group	T1 mean (SD)	T2 mean (SD)	ANOVA
STAI-S	Conventional	31.4 (8.8)	31.0 (9.9)	*F* = 0.4; *p* = 0.54 (group)
	Experimental	31.3 (8.0)	29.5 (8.8)	*F* = 3.3; *p* = 0.07 (time)
				*F* = 1.5; *p* = 0.23 (interaction)
CWS	Conventional	4.6 (1.5)	4.2 (1.4)	*F* = 2.7; *p* = 0.10 (group)
	Experimental	4.5 (1.6)	3.7 (1.1)	*F* = 14.1; ***p*** **<** **0.001** (time), ES = 0.41
				*F* = 2.0; *p* = 0.16 (interaction)
IES	Conventional	21.7 (13.9)	14.4 (13.1)	*F* = 6.6; ***p*** **=** **0.01** (group), ES = 0.36
	Experimental	14.8 (13.4)	9.9 (12.0)	*F* = 34.7; ***p*** **<** **0.001** (time), ES = 0.53
				*F* = 1.4; *p* = 0.25 (interaction)
RAND-36 MCS score	Conventional	49.7 (11.4)	52.5 (8.8)	*F* = 0.25; *p* = 0.62 (group)
	Experimental	49.3 (10.9)	54.3 (7.6)	*F* = 24.5; ***p*** **<** **0.001** (time), ES = 0.41
				*F* = 2.0; *p* = 0.16 (interaction)

*T1* at inclusion; *T2* after 1-year follow-up; *STAI*-*S* State-Trait Anxiety Inventory-State (range 20–80); *CWS* cancer worry scale (range 3–12); *IES* impact of event scale (range 15–75); *MCS* mental component summary (standardized mean 50); *F* F-statistic; *ES* effect size

Number (*n*) varies due to missing answers: STAI-S; *n* = 144 (75/69), CWS; *n* = 143 (74/69), IES; *n* = 116 (58/58), RAND-36; *n* = 149 (76/73). Level of significance *p* < 0.05, printed in bold

### Detection of Recurrences

Total recurrence rate at 1 year after diagnosis was 8.6 % in the CSG (*n* = 8) and 8.0 % in the ESG (*n* = 7, *p* = 0.89). Recurrences occurred as loco-regional or in-transit metastases, regional lymph nodes, second primary melanomas or distant disease. More recurred (6/15 = 40 %; CGS: *n* = 3, ESG: *n* = 3) than nonrecurred patients (25/152 = 16.4 %; CGS: *n* = 9, ESG: *n* = 16) paid extra outpatient clinic visits (*p* = 0.025). Eight of the 15 recurrences (53.3 %) were patient-detected and not physician-detected (CSG 62.5 %, ESG 42.9 %, *p* = 0.45). Seven of the eight self-detecting patients (87.5 %) performed self-inspection at least once a month, whereas in the physician-detected group this was 57.1 % (*p* = 0.35). Self-inspection was performed at least once a month by 78.4 % of the CSG and 65.3 % of the ESG at T2 (*p* = 0.23; Table 2).

### Cost Analysis

Total costs of the hospital based melanoma follow-up in the first year after primary excision, including detection and treatment of recurrences and all registered visits, was only calculated for the 79 patients treated at the UMCG. The total expense for the ESG (*n* = 38) was €15,871.11, with a mean of €417.66 per patient, and €31,240.67 for the CSG (*n* = 41), with a mean of €761.97 per patient. This demonstrates a mean cost reduction of 45 % (€344.31, 95 % CI 85.9-602.7, *p* = 0.01) per patient in the ESG. The differences in number of outpatient clinic visits, and the type of diagnostics and surgeries performed, are presented in Table 2. Expenses incurred for comorbidities or GP consultations were not taken into account in this calculation.

## Discussion

The MELFO study is the first randomized, clinical trial on the subject of follow-up frequency in AJCC stage IB-II melanoma patients. The results provide evidence that the frequency of follow-up visits in these melanoma patients can be reduced, because neither anxiety, cancer worry, stress response symptoms, and mental health, nor detection of recurrences and second primaries, were negatively affected by a reduced follow-up surveillance schedule. Besides, this is accompanied with 45 % cost reduction of overall melanoma care and outpatient clinic visits.

Patients’ mental well-being was similar in both groups or even better in the group with a reduced follow-up schedule. Specifically, levels of anxiety, cancer worry, and mental health-related quality of life were comparable in the study groups, and significantly reduced stress response symptoms were reported by the experimental group that received low-intensity follow-up surveillance. A possible explanation for this last finding might be that high-intensity follow-up surveillance can provoke stress rather than provide assurance. Mixed feelings of melanoma patients regarding follow-up have previously been described, with the majority of patients thinking follow-up visits were worthwhile, but half found them anxiety provoking also.^18^ Stress response symptoms and cancer worry decreased significantly over the first year of follow-up and patients’ mental well-being improved in both groups, possibly because patients became accustomed to having melanoma or due to the prolonged disease-free time after diagnosis and treatment. These results support our hypothesis that a reduced follow-up schedule does not negatively affect melanoma patients’ mental well-being.

The clinicopathologic characteristics of the MELFO study group are representative for the Dutch melanoma population.^31^ Recurrence rate after 12 months follow-up was approximately 9 % in both study groups. In literature, recurrence rates for AJCC stage IB-II patients are described from 18 to 45 % with a median time to detection of 28 months.^21^ Patient-detected recurrences for stage I-III melanoma are reported to be 60–75 %.^12^,^22^,^24^,^32^ Of the small number of recurrences and second melanomas in the first year after diagnosis in this study, slightly more than half was patient-detected (53 %). The proportion of patients performing self-inspection at least once a month was higher in the patient-detected group, emphasizing the importance of patient education in relation to the detection of recurrences.

Schedule satisfaction was high in both groups, suggesting patients might not have a preference for a certain surveillance schedule but rely on the recommendations of their clinician. Almost a third of the patients reported that they paid extra melanoma-related visits to the specialist or GP, demonstrating that some patients take action when they suspect a recurrence or experience anxiety, regardless of the assigned schedule.

As the prevalence of melanoma continues to rise, the intensity of surveillance strategies becomes important in the context of contemporary resource use. Melanoma follow-up is associated with a major financial burden.^32^,^33^ With the increasing cost-consciousness in current healthcare, the mean cost reduction of 45 % per patient per year found in the MELFO study is considerable.

This study was limited by the number of patients included. According to the power analysis 89 patients were needed in each study group; however, 87 were assigned to the ESG. Nevertheless, because no differences or trends were found between the groups, these two patients would not have made a significant difference. Also, the number of patients who completed all questions in the PROMs was less than required. However, refusal (13 %) and dropout (7 % for follow-up and 17 % for PROMS) rates were rather low. Lastly, calculation of costs was only possible of patients treated at a University Medical Center and may be slightly different from costs made in smaller hospitals.

Most current guidelines on follow-up frequency are based on low-level evidence, with unknown impact on patients’ mental well-being.^8^^,^^9^ Several potential benefits of reducing the existing frequency of follow-up visits for AJCC stage I-II melanoma patients have been proposed. According to these observational studies and in line with the present RCT, low-intensity surveillance strategies seem more efficient and do not appear to adversely affect patients’ clinical outcomes.^17^,^24^,^32^,^34^^–^36 A survey conducted among melanoma specialists in Australia concluded that extended intervals may even encourage patients to return immediately in case of a suspicious lesion, rather than waiting for their next scheduled appointment.^16^ All MELFO patients were educated about monthly self-inspection of the skin and regional lymph nodes, increasing patients’ ability to detect a possible recurrence or second primary.^12^,^23^,^37^ More patients suspecting a recurrence paid a visit outside of the assigned schedule than those not suspecting a recurrence, underlining the relevance of providing patient-education materials.^23^

## Conclusions

Stage-adjusted reduced follow-up surveillance for AJCC stage IB-II melanoma patients does not appear to affect adversely patients’ mental well-being and the detection of recurrences and is economically favorable compared to currently conducted high-intensity surveillance. These results suggest that lower-intensity surveillance may be safely recommended in evidence-based melanoma follow-up guidelines. Prolonged follow-up regarding the effect of a reduced surveillance schedule is necessary to strengthen this recommendation. In addition, all surveillance programs should emphasize the importance of patient education at diagnosis to increase the ability of patients to perform self-inspection of their skin and lymph node bearing areas for the timely detection of recurrences.

## Electronic Supplementary Material

Below is the link to the electronic supplementary material.

Supplementary material 1 (DOC 300 kb)

## References

[1] Arnold M, Holterhues C, Hollestein LM (2014). Trends in incidence and predictions of cutaneous melanoma across Europe up to 2015. J Eur Acad Dermatol Venereol..

[2] Leiter U, Eigentler T, Garbe C (2014). Epidemiology of skin cancer. Adv Exp Med Biol..

[3] Erdmann F, Lortet-Tieulent J, Schuz J, Zeeb H, Greinert R, Breitbart EW, Bray F (2013). International trends in the incidence of malignant melanoma 1953–2008–are recent generations at higher or lower risk?. Int J Cancer..

[4] Melanoma Skin Cancer, 2015. http://www.cancer.org/cancer/skincancer-melanoma/detailedguide/melanoma-skin-cancer-key-statistics.

[5] Surveillance, Epidemiology and End Results Program. Melanoma of the skin, 2015. http://seer.cancer.gov/statfacts/html/melan.html.

[6] Lin AY, Wang PF, Li H, Kolker JA (2012). Multicohort model for prevalence estimation of advanced malignant melanoma in the USA: an increasing public health concern. Melanoma Res..

[7] Speijers MJ, Francken AB, Hoekstra-Weebers JEHM, Bastiaannet E, Kruijff S, Hoekstra HJ (2010). Optimal follow-up for melanoma. Expert Rev Dermatol..

[8] Rueth NM, Cromwell KD, Cormier JN (2015). Long-term follow-up for melanoma patients: is there any evidence of a benefit?. Surg Oncol Clin N Am..

[9] Cromwell KD, Ross MI, Xing Y (2012). Variability in melanoma post-treatment surveillance practices by country and physician specialty: a systematic review. Melanoma Res..

[10] Bichakjian CK, Halpern AC, Johnson TM (2011). Guidelines of care for the management of primary cutaneous melanoma. American Academy of Dermatology. J Am Acad Dermatol..

[11] Melanoom, Landelijke Richtlijn, 2012. Versie: 2.0. http://www.oncoline.nl/melanoom.

[12] Dummer R, Hauschild A, Lindenblatt N, Pentheroudakis G, Keilholz U (2015). ESMO Guidelines Committee. Cutaneous melanoma: ESMO Clinical Practice Guidelines for diagnosis, treatment and follow-updagger. Ann Oncol..

[13] Garbe C, Paul A, Kohler-Spath H (2003). Prospective evaluation of a follow-up schedule in cutaneous melanoma patients: recommendations for an effective follow-up strategy. J Clin Oncol..

[14] Francken AB, Bastiaannet E, Hoekstra HJ (2005). Follow-up in patients with localised primary cutaneous melanoma. Lancet Oncol..

[15] Rychetnik L, McCaffery K, Morton R, Irwig L (2013). Psychosocial aspects of post-treatment follow-up for stage I/II melanoma: a systematic review of the literature. Psychooncology..

[16] Rychetnik L, McCaffery K, Morton RL, Thompson JF, Menzies SW, Irwig L (2013). Follow-up of early stage melanoma: specialist clinician perspectives on the functions of follow-up and implications for extending follow-up intervals. J Surg Oncol..

[17] Scally CP, Wong SL (2014). Intensity of follow-up after melanoma surgery. Ann Surg Oncol..

[18] Baughan CA, Hall VL, Leppard BJ, Perkins PJ (1993). Follow-up in stage I cutaneous malignant melanoma: an audit. Clin Oncol (R Coll Radiol)..

[19] Morton RL, Rychetnik L, McCaffery K, Thompson JF, Irwig L (2013). Patients’ perspectives of long-term follow-up for localised cutaneous melanoma. Eur J Surg Oncol..

[20] Ferrone CR (2005). Ben Porat L, Panageas KS, Berwick M, Halpern AC, Patel A, Coit DG. Clinicopathological features of and risk factors for multiple primary melanomas. JAMA..

[21] Francken AB, Shaw HM, Accortt NA, Soong SJ, Hoekstra HJ, Thompson JF (2007). Detection of first relapse in cutaneous melanoma patients: implications for the formulation of evidence-based follow-up guidelines. Ann Surg Oncol..

[22] Francken AB, Shaw HM, Thompson JF (2008). Detection of second primary cutaneous melanomas. Eur J Surg Oncol..

[23] Korner A, Coroiu A, Martins C, Wang B. Predictors of skin self-examination before and after a melanoma diagnosis: the role of medical advice and patient’s level of education. *Int**Arch Med.* 2013;6:8-7682-6-8. 10.1186/1755-7682-6-8 PMC3599942 23446040

[24] Francken AB, Accortt NA, Shaw HM (2008). Follow-up schedules after treatment for malignant melanoma. Br J Surg..

[25] Melanoom en oogmelanoom, 2015. https://www.kanker.nl/uploads/file_element/content/567/brochure-Melanoom_en_oogmelanoom.pdf.

[26] Spielberger CD, Gorsuch RL (2013). State-trait anxiety inventory for adults: instrument (adult form) and scoring guide.

[27] Lerman C, Trock B, Rimer BK, Boyce A, Jepson C, Engstrom PF (1991). Psychological and behavioral implications of abnormal mammograms. Ann Intern Med..

[28] van der Ploeg E, Mooren TT, Kleber RJ, van der Velden PG, Brom D (2004). Construct validation of the Dutch version of the impact of event scale. Psychol Assess..

[29] Hays RD, Morales LS (2001). The RAND-36 measure of health-related quality of life. Ann Med..

[30] Cohen J (1988). Statistical power analysis for the behavioral sciences.

[31] Hollestein LM, van den Akker SA, Nijsten T, Karim-Kos HE, Coebergh JW, de Vries E (2012). Trends of cutaneous melanoma in The Netherlands: increasing incidence rates among all Breslow thickness categories and rising mortality rates since 1989. Ann Oncol..

[32] Livingstone E, Krajewski C, Eigentler TK (2015). Prospective evaluation of follow-up in melanoma patients in Germany: results of a multicentre and longitudinal study. Eur J Cancer..

[33] Hengge UR, Wallerand A, Stutzki A, Kockel N (2007). Cost-effectiveness of reduced follow-up in malignant melanoma. J Dtsch Dermatol Ges..

[34] Turner RM, Bell KJ, Morton RL (2011). Optimizing the frequency of follow-up visits for patients treated for localized primary cutaneous melanoma. J Clin Oncol.

[35] Poo-Hwu WJ, Ariyan S, Lamb L (1999). Follow-up recommendations for patients with American Joint Committee on cancer stages I–III malignant melanoma. Cancer..

[36] Shumate CR, Urist MM, Maddox WA. Melanoma recurrence surveillance. Patient or physician based? *Ann Surg.* 1995;221:566–9; discussion 569–71. 10.1097/00000658-199505000-00014 PMC1234640 7748038

[37] Moore Dalal K, Zhou Q, Panageas KS, Brady MS, Jaques DP, Coit DG. Methods of detection of first recurrence in patients with stage I/II primary cutaneous melanoma after sentinel lymph node biopsy. *Ann Surg Oncol*. 2008;15:2206–14. 10.1245/s10434-008-9985-z 18512102

