# Supplementary material for: The MELFO-Study: Prospective, Randomized, Clinical Trial for the Evaluation of a Stage-adjusted Reduced Follow-up Schedule in Cutaneous Melanoma Patients—Results after 1 Year
Source: Ann Surg Oncol. 2016 May 18;23:2762–71. doi: 10.1245/s10434-016-5263-7 (PMC4972865; doi:10.1245/s10434-016-5263-7)
Supplement: Supplementary file 1 — Supplementary material 1 (DOC 300 kb) [file 10434_2016_5263_MOESM1_ESM.doc]

**PATIENT EDUCATION MATERIAL FOR MELANOMA PATIENTS**

1. THE FOLDER "Melanoma" OF THE DUTCH CANCER SOCIETY

https://www.kanker.nl/uploads/file_element/content/567/brochure-Melanoom_en_oogmelanoom.pdf

1. ADDITIONAL WRITTEN INFORMATION ABOUT SELF-INSPECTION

**SELF-INSPECTION**

The self-inspection you are advised to do after you have had a melanoma is twofold. Of importance are inspection on: a second (primary) melanoma and a recurrence (metastasis) of the removed melanoma. Information on self-inspection can be found on this sheet.

**A second melanoma**

People who once had a melanoma have an increased chance of getting a second (primary) melanoma compared to people who never had a melanoma. This probability lies between 3-8% and depends on skin type and environmental factors such as exposure to ultraviolet (UV) radiation, and (hereditary) predisposition. Because you know you have an increased chance of developing a second melanoma, it is wise to systematically monitor your skin for these malignant moles.

A melanoma can develop anywhere on the skin. Sometimes it arises from an existing mole, but it can also occur on a location with previously undamaged skin. For these reasons it is important to inspect the entire skin of your body and to do so with some regularity; for example, every two months.

Changes that may indicate melanoma:
- Mole which is thickening or enlarging

- Mole which darkens
- Mole that changes its shape, in particular if it develops a serrated edge
- Moles whose edge or surrounding is red
- Mole that itches
- Mole that bleeds
- Mole with a sore or scab

When melanoma occurs in the intact skin, it creates a new pigment spot, that will also exhibit the above symptoms.

Some people have many moles, making it difficult to determine changes in "that one mole". There are several ways to do this as good as possible:
1. Keeping a notebook; to exactly describe the moles that you want to keep an eye on; where they are, how big they are (overlay on blotting paper) and how they look. It is easier to determine a change the next time you check yourself.
2. Making pictures; once in a while you can take pictures of portions of your skin, which you can compare to the previous set of photos.

**Inspection of difficult skin areas**

There are areas of your skin that are very difficult to see by yourself (eg. the back). It is important to inspect these areas of the skin as well as the parts that you can see properly yourself. There are several ways you can do this.

**• You have someone who can help you inspect your skin**
If you have someone that can help you inspect your skin, it is best to ask the same person for help every time. By doing so, this person can detect changes the best.

**• You have no one who can help you inspect your skin**
If you have no one that can help you inspect your skin, you can use two mirrors, so you can inspect the back of your body (back and legs). It might be a bit of practice to get all parts of the skin in view right. When you have a lot of difficulty with performing this, you can ask your general practitioner whether it is necessary to get extra help inspecting the skin parts that are difficult to view for yourself. This would only be the case if you have an additional increased risk for the development of a second primary melanoma.

**A recurrence of the melanoma**

Depending on the type of melanoma that was diagnosed, you have a risk of developing a recurrence of the melanoma (a metastasis). Research has shown that most recurrences are discovered by patients themselves. This is because a recurrence of a melanoma is often visible on the skin or palpable under the skin or in the lymph nodes, and therefore often noticed by the patient between the planned checks in the hospital. This finding has led to a greater role for you as a patient in the detection of recurrences. For that reason, through this information you are instructed how you can best detect a melanoma recurrence.

**Where do recurrences occur?**

**Lymph nodes**

The first metastasis of a melanoma usually goes to the lymph nodes in the vicinity of the original melanoma. This will manifest itself by enlargement and/or solid feeling of one or more lymph nodes. However, if you have an infection, or a cold for example, these lymph nodes may also enlarge; this decreases after recovery from the inflammation. In case of a metastasis, the size will increase instead. If you are unsure whether it is an inflammatory lymph node or a malignant lymph node of the melanoma, you are advised to discuss this with your general practitioner.
The location of an expected lymph node metastasis, depending on the location of the removed melanoma, is indicated in Figure 2.


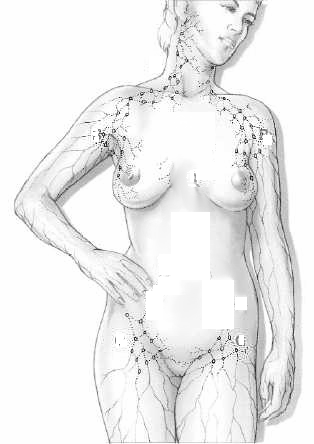


# **I**

# **II**

**II**

**III**

**III**

**IV**

**IV**

**Figure 2.** This figure shows the drainage areas of the lymph fluid. In particular, in the neck (I), armpits (II), in the elbow (III) and groin (IV) a recurrence of the melanoma would be expected, depending on the place where your melanoma has been removed.

**Local and intransit metastasis**

These are metastases arising between the scar of the removed melanoma and the regional lymph nodes that belong to it. These metastases are often small lumps in or under the skin with a size of a few millimeters to a few centimeters. If your melanoma was located on the back, the same measures as described above apply for the inspection of difficult areas of the skin (page 2).

**Distant metastasis**

Distant metastases can occur anywhere in the body. The most common place is the lungs. In general, it is important to listen to your body carefully. If you experience persistent symptoms that you are not used to, it is wise to consult your doctor to look whether a metastasis might be present indeed.
